# Changes in Physicochemical Characteristics and Antioxidant Activities of Dried Shiitake Mushroom in Dry-Moist-Heat Aging Process

**DOI:** 10.3390/foods12142714

**Published:** 2023-07-15

**Authors:** Supakit Chaipoot, Pairote Wiriyacharee, Rewat Phongphisutthinant, Srirana Buadoktoom, Aungkana Srisuwun, Chalermkwan Somjai, Sirasit Srinuanpan

**Affiliations:** 1 Multidisciplinary Research Institute, Chiang Mai University, Chiang Mai 50200, Thailand; 2 Center of Excellence in Microbial Diversity and Sustainable Utilization, Chiang Mai University, Chiang Mai 50200, Thailand; 3 Division of Product Development Technology, Faculty of Agro-Industry, Chiang Mai University, Chiang Mai 50100, Thailand; 4 Processing and Product Development Factory, The Royal Project Foundation, Chiang Mai 50100, Thailand; 5 Department of Biology, Faculty of Science, Chiang Mai University, Chiang Mai 50200, Thailand

**Keywords:** shiitake mushroom, amino acid profile, umami compounds, dry-moist-heat system, antioxidant activity

## Abstract

Shiitake mushrooms are prized for their unique flavor and bioactive properties. While there has been extensive research on drying methods, a comprehensive investigation of the effects of drying parameters in the dry-moist-heat system on shiitake quality is still needed. This study aimed to investigate the effects of dry-moist-heat aging on dried shiitake mushrooms comprehensively. Four aging temperatures, specifically 50, 60, 70, and 80 °C, were applied to the mushrooms, maintaining a constant humidity level of 75% RH and aging duration of 20 days. Color analysis revealed a progressive decrease in measured values as aging temperature increased, indicating noticeable changes in visual characteristics. Regarding amino acid composition, glutamic acid was found to be the predominant amino acid in shiitake mushrooms in the range of 90.29–467.42 mg/100 g. However, aging led to a reduction in overall amino acid content, with higher aging temperatures resulting in greater decline. Similarly, the equivalent umami content (EUC) also decreased (from 123.99 to 7.12 g MSG/100 g) with the increase in aging temperatures up to 80 °C, suggesting a decline in the overall umami taste sensation. Interestingly, despite the reduction in amino acid levels and umami content, the aging process positively impacted the phenolic compounds and the antioxidant activity of dried shiitake mushrooms. The antioxidative abilities of all aged mushroom extracts for DPPH, ABTS, and FRAP ranged from 65.01 to 81.39 µg TE/mL, 87.04 to 258.33 µg GAE/mL, and 184.50 to 287.68 µg FeSO_4_/mL, respectively. The utilization of aged temperature at 60 °C for 20 days with controlled relative humidity (~75%) should be a suitable aging condition of this edible mushroom with both antioxidant and umami qualities. Nevertheless, the control sample demonstrated higher levels of amino acid content and EUC compared to the aged samples. Conversely, the aged samples exhibited higher polyphenol content and greater antioxidant activity. Depending on specific requirements, these powders can be used in food formulation as flavor enhancers for control samples or as enriching agents for polyphenols and antioxidant activity in matured samples. Therefore, all of the powders obtained have potential applications in the field of nutrition.

## 1. Introduction

The shiitake mushroom (*Lentinus edodes*) is not only a widely consumed edible fungus but is also deeply rooted in the culinary traditions of various Asian countries. A comprehensive report by Data Bridge Market Research [1] reveals compelling insights into the global market for shiitake mushrooms, projecting its value to soar to an impressive figure of approximately USD 2300 million by the year 2029. This anticipated growth reflects a compound annual growth rate (CAGR) of 8.7% over the period from 2022 to 2029, highlighting the mushroom’s increasing popularity and market demand. One of the standout characteristics of shiitake mushrooms is their remarkable ability to enhance flavors, a quality attributed to the presence of both volatile and non-volatile components. These components include amino acids such as glutamic acid (Glu), aspartic acid (Asp), and monosodium glutamate (MSG), which contribute to the savory taste profile. Additionally, 5′-nucleotides, including 5′-guanosine monophosphate (5′-GMP), 5′-inosine monophosphate (5′-IMP), 5′-xanthosine monophosphate (5′-XMP), and 5′-adenosine monophosphate (5′-AMP) [2,3,4], further enhance the umami taste sensation. Moreover, specific umami amino acid sequences, namely Cys-Met, Glu-Pro-Glu, and Gly-Cys-Gly, play a significant role in the creation of the sought-after umami taste in shiitake mushrooms [2,3,4,5]. These compounds contribute to the overall sensory experience and desirability of shiitake mushrooms, making them a cherished ingredient in various culinary applications and food preparations.

During the drying process of shiitake mushrooms, the exposure to heat leads to the formation of volatile compounds, which become more pronounced as a result of the Maillard reaction and protein degradation. The Maillard reaction, a complex chemical process between amino acids and reducing sugars, generates a wide range of compounds responsible for the distinct aroma and flavor of dried shiitake mushrooms. Additionally, protein degradation contributes to the release of peptides and amino acids, further enhancing the overall flavor profile. As the mushrooms undergo drying, the reduction in moisture content allows for the concentration of these volatile compounds, intensifying their impact on the sensory characteristics of the dried mushrooms. The synthesis of various compounds, including hydrocarbons, aromatic compounds, and heterocyclic compounds, plays a pivotal role in creating the enticing burnt and caramel flavors associated with dried shiitake mushrooms. The rich flavor profile achieved through this drying process makes dried shiitake mushrooms highly versatile and suitable for a wide array of savory recipes. Their distinctive aroma and intensified flavors can elevate the taste of various dishes, adding depth and complexity. Therefore, dried shiitake mushrooms are valued not only for their nutritional content but also for their unique and savory flavor profile, which enhance culinary creations [2,6,7].

In addition to its their pleasant taste, shiitake mushrooms offer a wide range of nutrients and bioactive components, each harboring distinct biological properties and health-promoting benefits. This fungus is a rich source of diverse micro- and macronutrients, including polysaccharides, fiber, phenolics, ergosterol, minerals, and vitamins [5,8,9]. These nutritional elements contribute to the overall value of shiitake mushrooms as a wholesome food. Importantly, these compounds in shiitake mushrooms exhibit multifunctional properties, rendering them effective as antioxidants, anti-aging agents, antidiabetic agents, antitumor agents, antimicrobial agents, antiviral agents, antihyperlipidemic agents, anticholesterol agents, immunomodulators, and antihepatotoxic agents [9]. The diverse range of bioactive components present in shiitake mushrooms underscores their potential as a valuable natural resource for promoting health and well-being. Given their exceptional nutritional composition and health-enhancing properties, shiitake mushrooms find applications across a wide spectrum of consumable products. They serve as functional ingredients, adding both flavor and nutritional value to various food preparations. Additionally, shiitake mushrooms are utilized in fortifying foods to boost their nutritional content and are employed as dietary supplements to provide additional health benefits. Furthermore, the bioactive components found in shiitake mushrooms have garnered attention in the pharmaceutical industry, where they are explored for their potential in the development of therapeutic interventions [8]. These multifaceted applications further illustrate the significant value and versatility of shiitake mushrooms in diverse fields.

Dried shiitake mushrooms have gained wide popularity and are readily available, offering numerous advantages such as increased consumer preference, extended shelf life, and convenient distribution. These dried mushrooms have become a favored choice among consumers due to their enhanced flavor and versatility in various culinary applications. Additionally, the drying process significantly prolongs their shelf life, ensuring their availability for extended periods. Among the different methods employed for mushroom drying, hot-air drying is the most commonly used technique. This method is favored for its cost-effectiveness in terms of electricity consumption and its straightforward implementation, making it suitable for both small-scale and large-scale production [2]. Hot-air drying involves subjecting the mushrooms to controlled heated air, which facilitates the removal of moisture (less than or equal to 13% of water content) and results in the preservation of the mushroom’s characteristics, including taste, aroma, and nutritional value. During the drying process of mushrooms, a thermal reaction takes place, involving non-enzymatic glycosylation and the Maillard reaction. These reactions contribute to the formation of complex chemical compounds that are responsible for the distinctive flavor and aroma of dried shiitake mushrooms. By precisely controlling the temperature and maintaining a specific humidity level in the moist-dry-heat technique, the antioxidant activities of the mushrooms can be enhanced. This technique not only preserves the nutritional properties of the mushrooms but also promotes the formation of conjugated/complex compounds during prolonged storage, further enhancing their overall quality and health benefits [10,11]. The application of controlled drying methods, such as hot-air drying with precise temperature and humidity control, ensures that dried shiitake mushrooms retain their nutritional value, distinct flavors, and appealing aromas. This makes them highly suitable for various culinary purposes, as well as for use in food processing, where their concentrated flavor and extended shelf life offer practical advantages. Moreover, the preservation of antioxidant activities and the formation of complex compounds during the drying process contribute to the potential health benefits associated with consuming dried shiitake mushrooms.

Although there have been several studies investigating the effects of dry-moist-heat aging procedures on various dried mushrooms, there is a lack of information specifically regarding the impact on dried shiitake mushrooms. Especially, the impact of aging temperature during dry-moist-heat aging procedures on dried shiitake mushroom quality has rarely been reported. Hence, the principal objective of this study was to investigate the alterations in the physical attributes, amino acid composition, umami compounds, phenolic constituents, and antioxidant properties of dried shiitake mushrooms during the dry-moist-heat aging process. Multiple samples of mushrooms were exposed to different aging temperatures (50, 60, 70, and 80 °C) while meticulously controlling humidity levels and aging duration to maintain the integrity and precision of the findings.

## 2. Materials and Methods

### 2.1. Materials and Chemicals

Fresh shiitake mushrooms were sourced from the Royal Project Foundation in Chiang Mai, Thailand. Chemical substances and standards used in this study included 5′-guanosine monophosphate (Sigma-Aldrich, Bavaria, Germany), 5′-inosine monophosphate (Sigma-Aldrich, Jakarta, Indonesia), 5′-xanthosine monophosphate (ChemCruz, CA, USA), 2,2-diphenyl-1-picrylhydrazyl (Sigma-Aldrich, MA, USA), 1,4-dithiothreitol (Loba Chemie, Mumbai, India), boric acid (RCI Labscan, Bangkok, Thailand), N-acetyl-L-cysteine (Merck, Darmstadt, Germany), octanoic acid (Merck, Darmstadt, Germany), perchloric acid 70% (QRec, Auckland, New Zealand), O-phthalaldehyde (Sigma-Aldrich, Tokyo, Japan), Folin–Ciocalteu reagent (Merck, Darmstadt, Germany), sodium carbonate (QRec, Auckland, New Zealand), acetic acid (RCI Labscan, Bangkok, Thailand), acetonitrile HPLC grade (RCI Labscan, Bangkok, Thailand), potassium sulfate (RCI Labscan, Bangkok, Thailand), sodium hypochlorite (Loba Chemie, Mumbai, India), trichloroacetic acid (Supelco, Darmstadt, Germany), and tri-sodium citrate dehydrate (RCI Labscan, Bangkok, Thailand).

Amino acid mixtures (type H) consisting of 17 types of amino acids were purchased from Wako Pure Chemical Corporation in Osaka, Japan. HPLC-grade solvents, including acetonitrile and methanol, were obtained from RCI Labscan in Bangkok, Thailand. All solutions and dilutions were prepared using deionized water generated by a Milli-Q water system (Zeneer UP 900, Seoul, Republic of Korea). Phenolic standards, including gallic acid, theobromine, protocatechuic acid, p-hydroxybenzoic acid, catechin, chlorogenic acid, caffeine, vanillic acid, caffeic acid, syringic acid, epicatechin, vanillin, p-coumaric acid, ferulic acid, sinapic acid, rutin, myricetin, quercetin, and trans-cinnamic acid, were purchased from Sigma-Aldrich.

### 2.2. Dried Shiitake Mushroom Preparation

Fresh shiitake mushrooms were subjected to drying using a hot-air dryer (model UNE 600, Memmert, Germany) at a temperature of 80 °C for a duration of 20 h or until the moisture content reached the range of 8–10% (a suitable drying condition of shiitake from Royal project foundation report in 2021–2022). Subsequently, the dried mushrooms were carefully stored in polyethylene bags at a temperature of −18 °C to prevent any potential physicochemical alterations during storage. These dried shiitake mushrooms served as the control sample for subsequent analyses.

### 2.3. Dry-Moist-Heat Aging Conditions on Shiitake Mushroom and Extraction Method

Dried shiitake mushrooms weighing 100 g were carefully positioned within a desiccator, ensuring that the relative humidity was stabilized at approximately 75% by employing a saturated salt solution [10]. The samples were subjected to different aging conditions, specifically varying the temperature levels (50, 60, 70, and 80 °C) in an incubator (Heraeus model D-6450 Hanau, Hanau, Germany) while maintaining a fixed aging period of 20 days. The control sample in this study comprised dried shiitake mushrooms that were not subjected to the dry-moist-heat aging process. Subsequently, all samples were promptly packed in polyethylene bags under vacuum conditions to mitigate any potential alterations and stored at a temperature of −18 °C in a freezer until further analysis. Prior to analysis, the samples were meticulously processed into a powdered form.

For sample extract preparation, approximately 10 g of shiitake powder was employed and subsequently boiled with water at a ratio of 1:5 (*w*/*v*) for a duration of 10 min. Following the boiling process, the mixture underwent centrifugation at 8500 rpm for 5 min using a Hettich UNIVERSAL 320 R centrifuge (Andreas Hettich, Tuttlingen, Germany) to obtain the supernatant for subsequent analysis.

### 2.4. Measurement of Physical Characteristics of Aged Shiitake Mushroom

#### 2.4.1. Color Values

The color values (L *, C *, h) of shiitake powder were analyzed using a model CR-400 colorimeter (Konica, Minolta, Japan). The color space of L * indicates lightless, C * represents chroma, and h is the hue angle.

#### 2.4.2. Moisture Content and Water Activity (a_w_)

The moisture content was determined using method 934.06 (AOAC, 2010). The water activity was determined with a water activity meter (model AWC200, Novasina, Schwyz, Switzerland).

### 2.5. Total Protein Content Measurement

Method 992.23 (AOAC, 2010) of total crude protein content using Dumas combustion was selected for use in this research.

### 2.6. Amino Acid Profiles Determination

The determination of the 17 amino acids was conducted following the post-column reaction method of Shimadzu protocol with Na type as described by Somjai et al. [11]. A Shim-pack Amino-Na column with dimensions of 100 mm × 6.0 mm I.D. and 5 μm particle size (P/N: 228-18837-91, Shimadzu, Kyoto, Japan) was employed, coupled with a prominence RF-20A detector (Shimadzu, Kyoto, Japan). The mobile phases used were prepared as three distinct solutions: A, B, and C. Mobile phases A and B consisted of sodium citrate buffers with pH values of 3.23 and 10.0, respectively (mobile phase A: 19.6 g of trisodium citrate dihydrate, 70 mL of absolute ethanol, 14.3 mL of 70% perchloric acid, 0.1 mL of octanoic acid with total volume of 1 L; mobile phase B: 58.8 g of trisodium citrate dihydrate, 12.4 g of boric acid, 5.2 g of sodium hydroxide with total volume of 1 L), while mobile phase C comprised an aqueous solution of 0.2 M sodium hydroxide. For the precolumn derivatization of amino acids, reaction reagents were prepared using ortho-phthalaldehyde (OPA) and N-acetylcysteine. The running conditions involved maintaining a column oven temperature of 60 °C, a flow rate of 0.4 mL/min, and an injected sample volume of 10 µL.

### 2.7. 5′-Nucleotide Compounds Analysis and Equivalent Umami Concentration

The analysis of three 5′-nucleotide compounds, namely 5′-GMP, 5′-IMP, and 5′-XMP, was conducted using the HPLC technique described in the method developed by Harada-Padermo et al. [3]. A Shimadzu HPLC system equipped with an Inertsil ODS-3 column (250 mm × 4.6 mm; 5 µm particle size) from GL Science, Japan, was employed, coupled with a photo-diode array detector operating at a wavelength of 254 nm. The elution of compounds was achieved using a gradient elution method with two mobile phases. Mobile phase A consisted of a potassium phosphate buffer (50 mM KH_2_PO_4_) adjusted to pH 4.8, while mobile phase B comprised absolute methanol (HPLC grade). The gradient elution conditions were as follows: 0–5 min with 0% mobile phase B, 14–22.5 min with 10% mobile phase B, and 23–30 min with 0% mobile phase B. The column temperature was maintained at 30 °C, and the flow rate was set at 0.5 mL/min. A sample volume of 10 µL was injected for analysis.

An equivalent umami concentration can expressed as umami taste intensity that was determined by calculating the concentrations of amino acids (glutamic acid; Glu and aspartic acid; Asp) and 5′-nucleotides (5′-IMP, 5′-GMP, 5′-XMP) using Equation (1), proposed by Harada-Padermo et al. [3] and Mau [12]:

EUC (g MSG/100 g) = ∑ a_i_b_i_ + 1218(∑ a_i_b_i_) × (∑ a_j_b_j_) (1)

where EUC represents the umami taste intensity in terms of grams of monosodium glutamate (MSG) per 100 g of sample. a_i_ denotes the concentration (g/100 g) of Glu and Asp, a_j_ represents the concentration (g/100 g) of 5′-nucleotides, b_i_ corresponds to the relative umami concentration for each amino acid (Glu = 1, Asp = 0.077), b_j_ signifies the relative umami concentration for 5′-nucleotides (5′-IMP = 1, 5′-GMP = 2.3, 5′-XMP = 0.61), and 1218 denotes the synergistic constant based on the concentration of g/100 g used.

### 2.8. Total Phenolic Content and Phenolics Determination with HPLC

The determination of the total phenolic content was based on the method proposed by Wang et al. [13], with some modifications. Approximately 1 mL of shiitake extract was mixed with 0.5 mL of Folin–Ciocalteu reagent in a test tube, followed by the addition of 3 mL of 20% Na_2_CO_3_ solution. After a reaction time of 30 min, the mixture was diluted with 10 mL of deionized water and incubated for 15 min. The absorbance of the resulting solution was measured at a wavelength of 760 nm. The total phenolic content was calculated as milligrams of gallic acid equivalent per 100 g dry basis. For the analysis of phenolic compounds, HPLC (Shimadzu, Kyoto, Japan) equipped with an SPD-M20A Prominence Diode Array Detector (DAD) was employed. An Inertsil C18 column (250 × 4.6 mm, GL Sciences, CA, USA) was utilized for the test. Two mobile phases, namely mobile phase A (2% acetic acid in water) and mobile phase B (100% acetonitrile), were prepared. The method adapted from Liaudanskas et al. [14] was used for determining the phenolic compounds, employing a gradient elution condition. The gradient elution program involved the following proportions of mobile phase B: 0–5 min, 5–7%; 5–25 min, 7–8%; 25–27 min, 8–11%; 27–32 min, 11–12%; 32–44 min, 12–15%; 44–52 min, 15–15%; 52–53 min, 15–32%; 53–59 min, 32–32%; 59–63 min, 32–20%; 63–69 min, 20–90%; 69–74 min, 90–90%; 74–75 min, 90–5%; followed by stopping the run at 85 min. The flow rate of the mobile phase was set at 1 mL/min, and the column temperature was maintained at 30 °C. Prior to analysis, a 500 µL aliquot of the shiitake extract was diluted with 500 µL of absolute acetonitrile in a microcentrifuge tube and filtered through a 0.45 µm membrane. A 10 µL injection volume was used for the analysis. Nineteen types of phenolic standards, including gallic acid, theobromine, protocatechuic acid, p-hydroxybenzoic acid, catechin, chlorogenic acid, caffeine, vanillic acid, caffeic acid, syringic acid, epicatechin, vanillin, p-coumaric acid, ferulic acid, sinapic acid, rutin, myricetin, quercetin, and trans-cinnamic acid, were detected at 280 nm.

### 2.9. Determination of Antioxidant Activity

All samples were subjected to antioxidant activity analysis using three different methods: DPPH radical scavenging activity, ferric reducing antioxidant power (FRAP), and ABTS radical cation, as described in Somjai et al. [11].

For the DPPH method, a sample of shiitake extract (1 mL) was combined with 2 mL of 0.2 mM 2,2-diphenyl-1-picrylhydrazyl (DPPH) in 80% methanol. The mixture was vigorously mixed and kept at room temperature in a dark place for 30 min. The absorbance at 517 nm was measured using a UV–Vis spectrophotometer (UV1800; Shimadzu, Japan). A blank was prepared following the same procedure but using distilled water instead of the sample. A standard curve was constructed using Trolox (Sigma-Aldrich, Jakarta, Indonesia). The antioxidant activity was expressed as micrograms of Trolox equivalent (TE) per milliliter of extract.

For the ABTS method, an oxidant solution was prepared by mixing 2.45 mM K_2_S_2_O_8_ with 7 mM ABTS solution in 20 mM sodium acetate buffer (pH 4.5). The solution was then incubated at room temperature in the dark for 12–16 h to obtain a stable dark blue-green radical solution. The oxidant solution was diluted with 95% ethanol to an absorbance of 0.70 ± 0.02 at 734 nm, and this was used as the working solution. Next, 20 μL of the sample solution was added to 2 mL of the working solution, and the absorbance was measured at 734 nm after incubating the solution at room temperature in the dark for 6 min. The ABTS radical scavenging activity was calculated using a standard curve of gallic acid. The results were expressed as micrograms of gallic acid equivalent (GAE) per milliliter of shiitake extract.

For the FRAP method, the FRAP reagent solution was prepared by mixing 2.5 mL of 10 mM TPTZ solution in 40 mM HCl, 2.5 mL of 20 mM FeCl_3_·6H_2_O solution, and 20 mL of 300 mM acetate buffer (pH 3.6). The mixed solution was incubated at 37 °C for 30 min. Then, 50 μL of the extract sample was added to 750 μL of the FRAP solution and kept in the dark for 30 min. The change in solution color was measured at 593 nm. A standard curve was prepared using FeSO_4_·7H_2_O, and the results were expressed as micrograms of FeSO_4_ equivalent per milliliter of the extract.

### 2.10. Statistical Analysis

The statistical analyses were conducted using SPSS program version 17.0 (SPSS Inc., Chicago, IL, USA). Significance testing was performed using a one-way analysis of variance (ANOVA), followed by Tukey’s multiple comparisons test to assess differences between groups. A significance level of *p* ≤ 0.05 was employed to determine statistical significance.

## 3. Result and Discussion

### 3.1. Physical Characteristics of Aging Shiitake Mushroom

The results presented in Table 1 demonstrate noteworthy variations in the moisture content and water activity of the aging mushroom samples when compared to the control sample. Specifically, the moisture content ranged from 14.37% to 19.76%, while the water activity values fell within the range of 0.34 to 0.69. These increases were statistically significant, indicating that the aging process led to a significant uptake of moisture. However, it is important to highlight that the moisture content and water activity exhibited a slight decline when the aging temperature surpassed 70 °C. This suggests that higher temperatures might have caused the evaporation of moisture from the mushrooms, resulting in reduced water content.

The observation that the aging mushroom samples exhibited an a_w_ value above 0.6 can have implications for their durability. An a_w_ value above 0.6 suggests that there is sufficient moisture available in the samples, which could potentially contribute to chemical and microbial degradation over time [15]. In the context of powder durability, an a_w_ value above 0.6 is often considered a threshold for increased susceptibility to degradation. It indicates that the samples have a relatively high moisture content, creating a favorable environment for chemical reactions, enzymatic activity, and microbial growth [16]. These factors can lead to various forms of degradation, such as oxidation, hydrolysis, microbial spoilage, or changes in sensory attributes [15,16]. Based on existing literature, it is well-documented that samples with higher water activity levels are more prone to deterioration. The presence of moisture can facilitate chemical reactions, such as lipid oxidation and Maillard reactions, which can result in off-flavors, nutrient degradation, and color changes [17]. Additionally, higher water activity supports microbial growth and can lead to the proliferation of spoilage microorganisms or even pathogenic bacteria, thus reducing the shelf life and safety of the samples. Therefore, the a_w_ values above 0.6 observed in the aging mushroom samples suggest a potential vulnerability to degradation and necessitate proper storage conditions and packaging to minimize the impact of moisture. Mitigating moisture exposure, such as storing the samples in dry and airtight containers, employing desiccants, or utilizing appropriate packaging materials, is crucial to extend the durability and maintain the quality of the samples.

**Figure 1:**
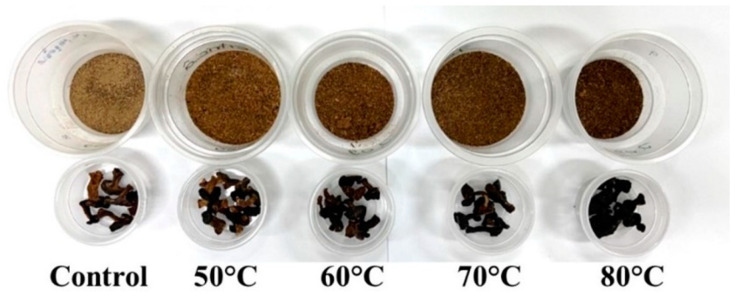
Shiitake mushroom with different aging temperatures.

To evaluate the color properties of the samples, including lightness (L *), chroma (C *), and hue angle (h), all treatments were subjected to analysis. The findings revealed a gradual decrease in the values of each color parameter as the aging temperature increased. Consequently, the mushroom powder underwent a notable transformation from a light brown hue in the control sample to a significantly darker black shade, as illustrated in Figure 1. The utilization of higher temperatures during the aging process appeared to contribute to the reduction in lightness, a phenomenon primarily attributed to the occurrence of the Maillard reaction. This complex chemical reaction is known to facilitate the development of brown pigments and is influenced by several factors, including temperature, moisture content, pH, and the ratio of amino acids to reducing sugars [10]. Additionally, edible mushrooms contain soluble sugars and polyols, such as glucose, mannitol, arabitol, arabinose, and trehalose, which are susceptible to degradation or conjugation during the heating process [18]. Consistent with previous studies conducted by Yang et al. [2] and Kantrong et al. [19], our investigation also observed an increase in the browning index value of dried shiitake mushrooms as the drying temperatures were elevated. This increase in browning index can be attributed to non-enzymatic browning reactions resulting from high thermal processes. It is important to note that, in addition to the Maillard reaction, alterations in the color parameters of dried mushroom products can occur due to non-enzymatic browning reactions, pigment degradation, oxidation of ascorbic acid, and other contributing factors [20,21,22]. These multiple mechanisms may interact and influence the overall color changes observed in the dried shiitake mushrooms during the aging process.

### 3.2. Total Protein and Free Amino Acids Content of Aging Shiitake Mushrooms

The investigation encompassed an in-depth assessment of the total protein content in each sample of shiitake mushrooms, both before and after the aging process. Surprisingly, the aging process did not yield substantial variations in the quantity of protein among the shiitake samples, indicating that the aging temperature did not significantly impact the overall protein content. The protein content for all treatments fell within a narrow range of 24.62% to 28.68%, suggesting a consistent protein composition across the samples. Shifting the focus to the free amino acid content of each aged sample (refer to Table 2), it was observed that four types of amino acids, namely isoleucine, leucine, tyrosine, and arginine, were not detected in any of the shiitake mushroom samples, regardless of the aging temperature applied. Prior to the aging process, the dried mushrooms exhibited significantly higher levels of specific amino acids compared to the aged samples. However, exceptions were noted for certain amino acids, including aspartic acid, alanine + cysteine, methionine, phenylalanine, and lysine, where no significant differences were observed between the dried mushrooms and the aged samples. Among the identified amino acids, glutamic acid emerged as the most prominent, with an average content of approximately 467.42 mg per 100 g of shiitake mushrooms. Other amino acids detected in substantial amounts in these mushrooms included valine, alamine + cysteine, lysine, phenylalanine, threonine, serine, glycine, aspartic acid, proline, histidine, and methionine. 

Significantly, the aging process led to a notable reduction in the quantity of several amino acids, particularly threonine, serine, glutamic acid, proline, glycine, valine, and histidine, as the aging temperature increased. These amino acids experienced a diminishing trend with elevated aging temperatures, suggesting their susceptibility to degradation or modification during the aging process. Conversely, certain amino acids, such as aspartic acid, alanine + cysteine, methionine, phenylalanine, and lysine, exhibited slight changes in their content, but these differences were not statistically significant (*p* > 0.05). The decline in amino acid content observed during the aging process can be attributed to the interaction between free amino acids and reducing sugars through non-enzymatic Maillard reactions, which are known to occur under high-temperature conditions [21,23]. Furthermore, various non-covalent interactions between amino acids and phenolics may contribute to the formation of conjugated compounds, which could also influence the observed changes in amino acid content. These interactions, including hydrophobic interactions, van der Waals forces, electrostatic interactions, and hydrogen bonding, have been reported to affect the stability and composition of amino acids during food processing and storage [10,11,24,25].

### 3.3. Compounds of 5′-Nucleotides and Equivalent Umami Content (EUC) of Aged Shiitake Mushroom

**Figure 2:**
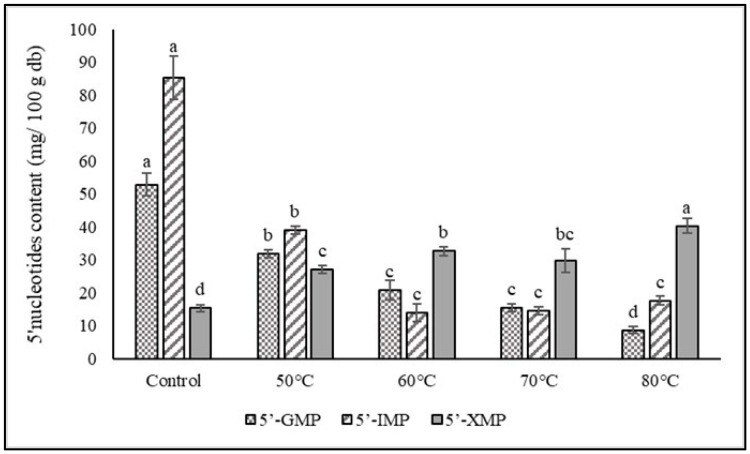
5′-nucleotide compounds (5′-GMP, 5′-IMP, and 5′-XMP) of dried shiitake mushroom at different aging temperatures. Different lowercase letters indicate significant differences among the different aging temperatures in 5′-nucleotide compounds levels (*p* < 0.05).

**Figure 3:**
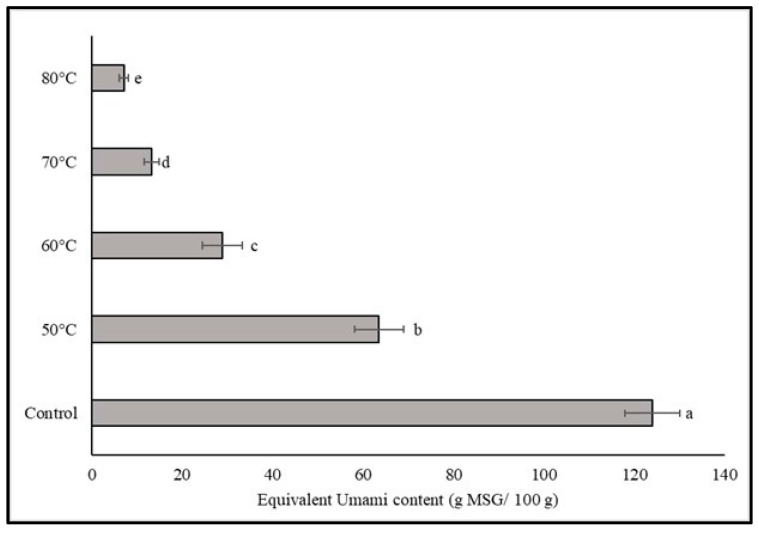
EUC values of dried shiitake mushroom at different aging temperatures. Different lowercase letters indicate significant differences among the different aging temperatures in EUC values (*p* < 0.05).

In this study, the impact of a dry-moist-heat aging process on the levels of three 5′-nucleotide compounds (5′-GMP, 5′-IMP, and 5′-XMP) in dried shiitake mushrooms was investigated, as illustrated in Figure 2. The results revealed that the dried mushrooms that did not undergo the aging process displayed the highest levels of two 5′-nucleotides, namely 5′-GMP and 5′-IMP, at 52.77 and 85.25 mg/100 g, respectively. However, with an increase in incubation temperature, the quantities of these two nucleotides gradually decreased, ranging from 31.87 to 8.62 mg/100 g for 5′-GMP and 38.98 to 13.92 mg/100 g for 5′-IMP. Notably, when the aging temperature exceeded 60 °C, the level of 5′-IMP remained relatively stable, while the content of 5′-GMP significantly decreased after aging at 80 °C. Furthermore, the highest level of 5′-XMP was found in the aged shiitake mushrooms incubated at 80 °C, indicating that higher temperatures slightly enhanced the content of this specific nucleotide, ranging from 15.29 to 40.25 mg/100 g. The equivalent umami content (EUC) values, presented in Figure 3, represent the concentration of monosodium glutamate (MSG)-like acids (aspartic acid and glutamic acid) and umami 5′-nucleotides. The findings demonstrated that all the aging mushroom samples exhibited a significant reduction in the EUC value as the aging temperature increased. The EUC values ranged from 7.12 to 123.99 g MSG/100 g, indicating a decrease in the intensity of umami taste. This value was correlated to the amount of two amino acids (Glu and Asp), including 5′-nucleotides, and the aged shiitake also exhibited these components in smaller quantities. The aging process with an overheating condition could decrease the EUC value by more than 90% compared to a control treatment.

According to Zhao et al. [26], each free amino acid can be categorized based on its contribution to different taste characteristics. Umami taste primarily arises from aspartic acid and glutamic acid, while the bitter taste is influenced by seven amino acids, including histidine, arginine, valine, methionine, isoleucine, leucine, and phenylalanine. Additionally, serine, glycine, threonine, alanine, and proline are associated with sweetness, while cysteine, tyrosine, and lysine are characterized as tasteless. Thus, the findings of this study indicate that the aging process at excessively high temperature levels may reduce the bitter taste due to the depletion of certain amino acids that contribute to the characteristic bitterness of these mushrooms, thereby rendering them more palatable. However, the umami and sweet tastes were observed to decline due to the overheating process, which could result in the degradation of 5′-nucleotides or the loss of umami flavor [15,18,26,27]. In addition, the flavor of dried shiitake mushroom was related to the volatile components due to the aldehydes derived from the oxidation and degradation of unsaturated fatty acids. Strecker degradation from the Maillard reaction also promoted the content of aldehydes through the drying process [28]. 

### 3.4. Phenolic Compounds Found in Aged Shiitake Mushroom

**Figure 4:**
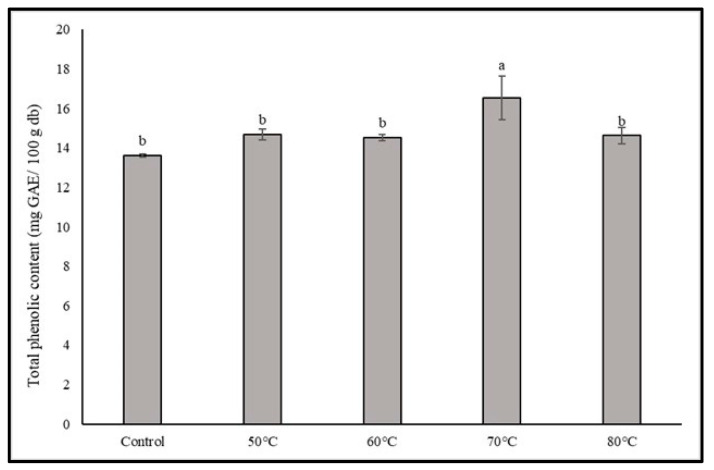
Total phenolic content of dried shiitake mushroom at different aging temperatures. Different lowercase letters indicate significant differences among the different aging temperatures in total phenolic content (*p* < 0.05).

The findings presented in Figure 4 indicate the content of total phenolic compounds in dried shiitake mushrooms subjected to different aging temperatures. The observed range of 13.61–16.53 mg GAE/100 g for total phenolic content suggests that all shiitake treatments contained significant levels of phenolic compounds. Notably, the aging temperature of 70 °C resulted in the highest level of phenolic compounds among the tested temperatures. These results suggest that the aging process can impact the phenolic composition of dried shiitake mushrooms. The composition of phenolic compounds in the dried mushroom samples was further investigated using HPLC, as shown in Table 3. The analysis revealed the presence of six phenolic compounds consistently present in all dried mushroom samples, namely vanillic acid, p-hydroxybenzoic acid, theobromine, chlorogenic acid, syringic acid, and trans-cinnamic acid. However, it should be noted that 13 other types of phenolics were not detected in these samples, indicating variations in the phenolic profiles of dried shiitake mushrooms. The concentrations of the identified phenolic compounds varied across the shiitake treatments. Theobromine, p-hydroxybenzoic acid, chlorogenic acid, vanillic acid, syringic acid, and trans-cinnamic acid were present at different levels. For instance, theobromine ranged from 38.79 to 78.80 µg/100 g, p-hydroxybenzoic acid ranged from 21.97 to 56.84 µg/100 g, chlorogenic acid ranged from 9.34 to 36.91 µg/100 g, vanillic acid ranged from 90.15 to 50.87 µg/100 g, syringic acid ranged from 8.34 to 11.55 µg/100 g, and trans-cinnamic acid ranged from 0.00 to 14.49 µg/100 g. These findings suggest that the aging process, particularly at higher temperatures, can influence the levels of specific phenolic compounds, resulting in their increase.

However, it is important to consider that high temperatures during the aging process can also lead to a reduction in the quantity of certain phenolic compounds. This reduction could potentially be attributed to heat-induced changes, which may result in the degradation or alteration of phenolic compounds. The free form of these compounds could release and increase as the temperature and heating time increase [29]. Previous studies by Ren et al. [21] and Dong et al. [30] have demonstrated the complex effects of thermal processing on phenolic compounds, highlighting that the impact can vary depending on the mushroom’s cell structure. Thus, the observed variations in phenolic profiles in dried shiitake mushrooms can be attributed to a combination of factors, including the specific aging temperature, the structural composition of the mushrooms, and the intricate nature of the heat-induced processes affecting the phenolic compounds. Moreover, it is worth noting that variations in the content and composition of phenolic compounds have been reported in shiitake mushrooms by Wang et al. [13]. These variations may arise from factors such as the geographical location of mushroom cultivation, mushroom variety, maturity stage, production processes employed, and the extraction methods used to analyze phenolic compounds. Therefore, the specific characteristics of the mushrooms and the parameters of the aging process can contribute to the observed differences in phenolic profiles among different studies.

### 3.5. Antioxidant Activities of Aged Shiitake Mushroom

**Figure 5:**
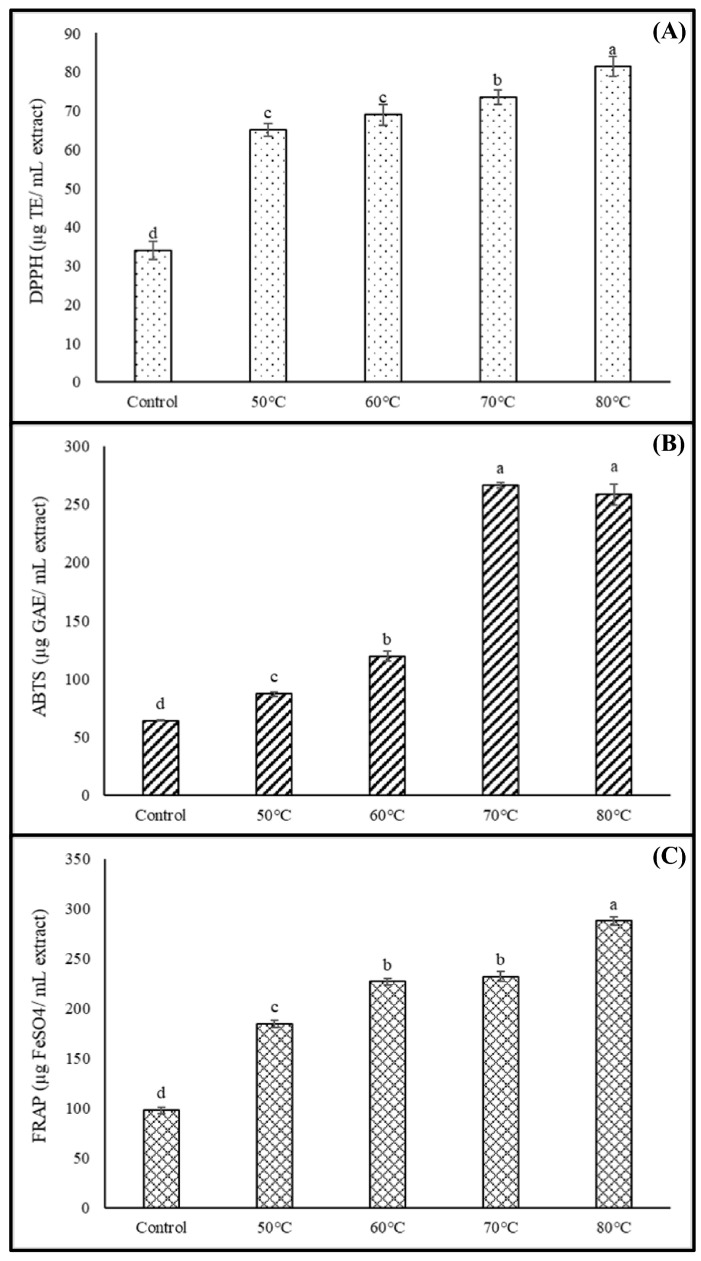
Antioxidant activities for (**A**) DPPH, (**B**) ABTS, (**C**) FRAP methods of dried shiitake mushroom at different aging temperatures. Different lowercase letters indicate significant differences among the different aging temperatures in antioxidant activities (*p* < 0.05).

The findings presented in Figure 5 provide valuable insights into the antioxidative ability of dried shiitake mushrooms, as assessed through the DPPH, ABTS, and FRAP methods. The results clearly demonstrate a positive relationship between aging temperature and antioxidant activity. Specifically, both DPPH and FRAP values exhibited a gradual increase as the incubation temperature rose. The antioxidant activities ranged from 65.01 to 81.39 µg TE/mL for DPPH and 184.50 to 287.68 µg FeSO_4_/mL for FRAP in the aged shiitake mushroom extracts. It is noteworthy that the highest levels of antioxidant activity were observed in the mushrooms aged at 80 °C, displaying approximately 2.4-fold higher DPPH value and 2.9-fold higher FRAP value compared to the untreated mushrooms. Similarly, the ABTS radical scavenging activity of the aged shiitake extract reached its peak value (266.27 µg GAE/mL) when incubated at temperatures above 70 °C, demonstrating an approximately four-fold increase compared to the control sample.

Similar results were observed in a study on black garlic that underwent an aging process at a regular temperature of 70 °C and humidity of 80%, wherein an increase in the levels of total antioxidant capacity, DPPH scavenging, and reducing power was observed [31]. The observed increase in antioxidant activity can be attributed to the formation of various compounds during the excessive thermal processing involved in the aging procedure. These compounds include Maillard reaction products and conjugated compounds, which have been previously identified as contributors to the oxidative capacity of food. Specifically, radical chain reactions involving pyrrole and hydroxyl groups contribute to the antioxidative potential of these compounds. Moreover, the conjugated substances present in the aged shiitake mushrooms exhibit properties such as scavenging reactive oxygen species, chelating metal ions, degrading hydrogen peroxide, and facilitating electron transfer. These mechanisms, discussed in studies by Somjai et al. [10], Wang et al. [32], Hamdani et al. [33], and Nooshkam et al. [34], contribute to the overall antioxidative capacity observed in the aged shiitake mushrooms. The Maillard reaction can exert both advantageous and unfavorable impacts on the physicochemical, biological, and sensory attributes of food products [10]. To prevent the formation of undesired components, this reaction necessitates careful management of various parameters, including incubation time, pH, a_w_, and temperature. Therefore, it is essential to subject the Maillard reaction products to rigorous safety assessments before their consumption [35,36]. Furthermore, the high heating conditions during the aging process have an additional impact on the antioxidant properties of the mushrooms. The heat-induced destruction of the mushroom’s cell wall structure facilitates the release of bioactive compounds during the subsequent extraction process. This structural disruption enhances the accessibility of these bioactive compounds, further contributing to the observed antioxidant activity. The influence of cell wall structure on the extraction of bioactive compounds has been acknowledged in previous study by Piskov et al. [20].

## 4. Conclusions

Based on the findings of this study, it can be observed that the physicochemical qualities and antioxidative activity of dried shiitake mushrooms underwent significant changes as a result of the dry-moist-heat aging process. These changes were particularly pronounced at higher aging temperatures. The outcomes of this study lay the groundwork for future investigations into the enhancement of antioxidant activity in dried mushrooms through controlled aging processes, considering factors such as relative humidity, temperature, time, and composition of raw material. However, it is worth noting that certain amino acid components and umami compounds exhibited a decline in their concentrations, likely due to excessive thermal treatment. These alterations may have implications for the sensory attributes of the mushrooms. However, this approach under appropriate conditions could provide useful information for several agricultural products to enhance their bioactivity, especially antioxidant properties, with negligible damage to the raw material. In this case of dried shiitake mushrooms, the utilization of an aged temperature of 60 °C for 20 days with controlled relative humidity (~75%) should be a suitable aging condition for this edible mushroom with both antioxidant and umami qualities. Nevertheless, the control sample demonstrated higher levels of amino acid content and EUC compared to the aged samples. Conversely, the aged samples exhibited higher polyphenol content and greater antioxidant activity. Depending on specific requirements, these powders can be used in food formulation as flavor enhancers for control samples or as enriching agents for polyphenols and antioxidant activity in matured samples. Therefore, all of the powders obtained have potential applications in the field of nutrition.

## Figures and Tables

**Table 1:** Moisture content, a_w_, and color values of shiitake mushroom at different aging temperatures.

Aging Condition		Control	50 °C	60 °C	70 °C	80 °C
Moisture content (%)		8.64 ± 1.95 ^c^	19.76 ± 1.88 ^a^	19.69 ± 0.30 ^a^	14.37 ± 0.21 ^b^	15.09 ± 0.13 ^b^
a_w_		0.34 ± 0.05 ^c^	0.69 ± 0.01 ^a^	0.68 ± 0.02 ^a^	0.61 ± 0.01 ^b^	0.62 ± 0.01 ^b^
Color values	L *	55.70 ± 2.60 ^a^	48.53 ± 1.51 ^b^	45.46 ± 1.84 ^cd^	46.42 ± 0.82 ^c^	44.54 ± 1.36 ^d^
	C *	13.92 ± 1.03 ^a^	12.75 ± 1.05 ^b^	12.01 ± 0.67 ^c^	10.74 ± 0.34 ^d^	8.72 ± 0.46 ^e^
	h	52.41 ± 2.82 ^a^	49.55 ± 2.00 ^b^	43.87 ± 1.44 ^c^	42.51 ± 0.81 ^c^	38.12 ± 1.68 ^d^

Data represented as mean ± SD of three replicates; ^a–e^ mean values within each row with different superscript letters were significantly different (*p* ≤ 0.05); control was the dried shiitake mushroom without aging. L * indicates lightness, C * represents chroma, and h is the hue angle.

**Table 2:** Composition of amino acids found in dried shiitake mushrooms at different aging temperatures.

Amino Acid (mg/100 g db)	Aging Condition (°C)				
	Control	50	60	70	80
Asp ^ns^	61.48 ± 18.98	51.05 ± 4.87	45.77 ± 2.04	39.51 ± 2.19	41.92 ± 5.21
Thr	127.81 ± 9.17 ^a^	90.19 ± 2.66 ^b^	61.64 ± 2.18 ^c^	38.20 ± 7.34 ^d^	43.11 ± 4.69 ^d^
Ser	98.85 ± 20.30 ^a^	111.60 ± 10.71 ^a^	90.81 ± 4.21 ^ab^	68.91 ± 3.94 ^bc^	54.40 ± 6.64 ^c^
Glu	467.42 ± 51.76 ^a^	399.08 ± 30.89 ^a^	283.32 ± 7.78 ^b^	155.25 ± 6.00 ^c^	90.29 ± 9.49 ^c^
Pro	45.83 ± 6.08 ^a^	42.67 ± 0.56 ^ab^	36.88 ± 1.61 ^bc^	30.29 ± 0.73 ^cd^	24.66 ± 1.22 ^d^
Gly	84.41 ± 12.99 ^a^	62.61 ± 2.63 ^b^	47.85 ± 0.25 ^bc^	37.57 ± 0.59 ^c^	34.43 ± 2.76 ^c^
Ala + Cys ^ns^	211.16 ± 34.33	222.06 ± 9.44	210.74 ± 0.66	193.02 ± 0.03	207.56 ± 12.28
Val	330.13 ± 28.62 ^ab^	354.37 ± 8.88 ^a^	337.35 ± 3.96 ^ab^	295.49 ± 16.65 ^bc^	286.14 ± 10.70 ^c^
Met ^ns^	8.42 ± 2.11	7.49 ± 1.45	8.18 ± 0.27	6.31 ± 0.61	4.01 ± 0.62
Ile	ND	ND	ND	ND	ND
Leu	ND	ND	ND	ND	ND
Tyr	ND	ND	ND	ND	ND
Phe ^ns^	152.51 ± 32.20	158.96 ± 13.03	157.89 ± 4.05	151.09 ± 4.11	135.12 ± 15.36
His	31.46 ± 1.94 ^a^	14.59 ± 0.93 ^b^	11.11 ± 1.30 ^c^	9.28 ± 1.18 ^c^	11.14 ± 0.46 ^c^
Lys ^ns^	197.15 ± 11.74	174.97 ± 29.04	152.66 ± 62.33	120.06 ± 61.55	143.10 ± 1.11
Arg	ND	ND	ND	ND	ND

Data represented as mean ± SD of three replicates; ^a–d^ mean values within each row with different superscript letters were significantly different (*p* ≤ 0.05); ND = not detected; ns = not significant; db = dry basis; control was the dried shiitake mushroom without aging; Asp = aspartic acid, Thr = threonine; Ser = serine; Glu = glutamic acid, Pro = proline; Gly = glycine; Ala + Cys = alanine + cysteine; Val = valine; Met = methionine; Ile = isoleucine; Leu = leucine; Tyr = tyrosine; Phe = phenylalanine; His = histidine; Lys = lysine; Arg = arginine.

**Table 3:** Composition of phenolics found in dried shiitake mushroom at different aging temperatures.

Phenolic Compound (µg/100 g db)	Aging Condition				
	Control	50 °C	60 °C	70 °C	80 °C
Gallic acid	ND	ND	ND	ND	ND
Theobromine	38.79 ± 5.46 ^c^	49.60 ± 0.85 ^b^	51.18 ± 4.35 ^b^	55.65 ± 1.56 ^b^	74.80 ± 0.11 ^a^
Protocatechuic acid	ND	ND	ND	ND	ND
*p*-Hydroxybenzoic acid	56.84 ± 2.76 ^a^	47.48 ± 3.44 ^b^	50.21 ± 1.78 ^b^	49.18 ± 1.95 ^b^	21.97 ± 0.39 ^c^
Catechin	ND	ND	ND	ND	ND
Chlorogenic acid	36.91 ± 0.14 ^a^	21.19 ± 5.28 ^b^	22.79 ± 1.09 ^b^	21.06 ± 0.68 ^b^	9.34 ± 0.78 ^c^
Caffeine	ND	ND	ND	ND	ND
Vanillic acid	50.87 ± 0.90 ^d^	62.70 ± 3.37 ^c^	73.05 ± 4.64 ^b^	90.15 ± 1.75 ^a^	65.72 ± 3.25 ^c^
Caffeic acid	ND	ND	ND	ND	ND
Syringic acid	8.70 ± 0.77 ^c^	10.04 ± 0.83 ^b^	11.14 ± 0.36 ^a^	11.55 ± 0.05 ^a^	8.34 ± 0.49 ^c^
Epicatechin	ND	ND	ND	ND	ND
Vanillin	ND	ND	ND	ND	ND
*p*-Coumaric acid	ND	ND	ND	ND	ND
Ferulic acid	ND	ND	ND	ND	ND
Sinapic acid	ND	ND	ND	ND	ND
Rutin	ND	ND	ND	ND	ND
Myricetin	ND	ND	ND	ND	ND
Quercetin	ND	ND	ND	ND	ND
*Trans*-cinnamic acid	TR	14.05 ± 1.05 ^a^	14.49 ± 1.94 ^a^	13.97 ± 0.11 ^a^	11.11 ± 0.14 ^b^

Data represented as mean ± SD of three replicates; ^a–d^ mean values within each row with different superscript letters were significantly different (*p* ≤ 0.05); ND = not detected; TR = trace amount; db = dry basis; control was the dried shiitake mushroom without aging.

## Data Availability

The original contributions presented in the study are included in the article. Further inquiries can be directed to the corresponding authors.
